# How adolescents’ lives were disrupted over the course of the COVID-19 pandemic: A longitudinal investigation in 12 cultural groups in 9 nations from March 2020 to July 2022

**DOI:** 10.1017/S0954579423001621

**Published:** 2024-01-26

**Authors:** W. Andrew Rothenberg, Ann T. Skinner, Jennifer E. Lansford, Dario Bacchini, Marc H. Bornstein, Lei Chang, Kirby Deater-Deckard, Laura Di Giunta, Kenneth A. Dodge, Sevtap Gurdal, Daranee Junla, Qin Liu, Qian Long, Paul Oburu, Concetta Pastorelli, Emma Sorbring, Laurence Steinberg, Liliana Maria Uribe Tirado, Saengduean Yotanyamaneewong, Liane Peña Alampay, Suha M. Al-Hassan

**Affiliations:** 1 Duke University, Durham, NC, USA; 2 University of Miami Miller, School of Medicine, Miami, FL, USA; 3 University of Naples “Federico II,”, Napoli, Italy; 4 NICHD, Bethesda, MD, USA; 5 UNICEF, New York, NY, USA; 6 Institute for Fiscal Studies, London, UK; 7 University of Macau, Taipa, China; 8 University of Massachusetts Amherst, Amherst, MA, USA; 9 Helsinki Collegium for Advanced Studies, Helsinki, Finland; 10 Università di Roma “La Sapienza,”, Rome, Italy; 11 University West, Trollhättan, Sweden; 12 Chiang Mai University, Chiang Mai, Thailand; 13 Chongqing Medical University, Chongqing, China; 14 Duke Kunshan University, Kunshan, China; 15 Maseno University, Maseno, Kenya; 16 Temple University, Philadelphia, PA, USA; 17 King Abdulaziz University, Jeddah, Saudi Arabia; 18 Universidad de San Buenaventura, Medellin, Colombia; 19 Ateneo de Manila University, Manila, Philippines; 20 Abu Dhabi Early Childhood Authority, Abu Dhabi, United Arab Emirates; 21 Hashemite University, Zarqa, Jordan

**Keywords:** COVID-19, adolescence, risk factors, longitudinal, cross-cultural

## Abstract

It is unclear how much adolescents’ lives were disrupted throughout the COVID-19 pandemic or what risk factors predicted such disruption. To answer these questions, 1,080 adolescents in 9 nations were surveyed 5 times from March 2020 to July 2022. Rates of adolescent COVID-19 life disruption were stable and high. Adolescents who, compared to their peers, lived in nations with higher national COVID-19 death rates, lived in nations with less stringent COVID-19 mitigation strategies, had less confidence in their government’s response to COVID-19, complied at higher rates with COVID-19 control measures, experienced the death of someone they knew due to COVID-19, or experienced more internalizing, externalizing, and smoking problems reported more life disruption due to COVID-19 during part or all of the pandemic. Additionally, when, compared to their typical levels of functioning, adolescents experienced spikes in national death rates, experienced less stringent COVID-19 mitigation measures, experienced less confidence in government response to the COVID-19 pandemic, complied at higher rates with COVID-19 control measures, experienced more internalizing problems, or smoked more at various periods during the pandemic, they also experienced more COVID-19 life disruption. Collectively, these findings provide new insights that policymakers can use to prevent the disruption of adolescents’ lives in future pandemics.

The lives of adolescents around the world continue to be disrupted by COVID-19, as the prevalence of anxiety and depressive disorders in youth have increased (World Health Organization, 2022), and more than one in three youth reported poor mental health during the COVID-19 pandemic (Centers for Disease Control and Prevention, 2022). As the pandemic wore on, developmental scientists identified that the pandemic disrupted adolescents’ lives by upending their daily routines (e.g., lockdowns and social isolation), disrupting their transitions to and through secondary school and higher education (e.g., virtual schooling) and disrupting relationships with their family members and peers (due to pandemic-related stress and sickness; Angela et al., 2022; Kauhanen et al., 2022; Manchia et al., 2022; Samji et al., 2022; Skinner et al., 2021). The current investigation evaluates adolescents’ perceptions of how disruptive the COVID-19 was to their lives, including to their daily routines, work, school, and family life, from March 2020 to July 2022.

Myriad risk factors have been identified as contributing to adolescents’ life disruption during COVID, ranging from COVID’s effects on the society an adolescent lives in, to the loss of family members during COVID, to adolescent confidence in, and compliance with, government regulations, to deteriorating adolescent mental health and behavior problems (Angela et al., 2022; Bornstein, 2021; Kauhanen et al., 2022; Manchia et al., 2022; Samji et al., 2022). However, developmental and prevention scientists still lack a complete understanding of how these myriad risk factors have disrupted adolescents’ lives over the course of the pandemic. Systematic reviews attribute this incomplete knowledge base to three gaps in existing literature.

First, virtually all existing work examining the disruptive effects of COVID-19 is cross-sectional and monocultural, so we still do not know how disruptive the COVID-19 pandemic has been to adolescents over the entirety of the pandemic in different cultures. Second, studies almost exclusively investigate how single risk factors impact how disruptive the COVID-19 pandemic has been to adolescents. so we do not know which constellation of risk factors might emerge as especially important to predicting COVID-19 disruption. Third, studies have not disaggregated between- and within-person predictors of COVID-19 disruption, so we cannot identify who among adolescents is most likely to be disrupted by COVID-19 across the entire course of the pandemic (a between-person effect) and when over the course of the pandemic spikes in particular risk factors are especially likely to lead to greater adolescent life disruption (a within-person effect). This 5-wave longitudinal investigation of 1,080 adolescents^1^ from 12 cultural groups in 9 nations who were 19 years old, on average, at the beginning of the pandemic seeks to fill all three of these gaps in existing literature. In so doing, it provides a comprehensive examination of risk factors that predict who among adolescents have experienced the most life disruption due to COVID-19 and when such disruptions were most likely to occur.

## Moving from single-culture cross-sectional studies to multiculture longitudinal analyses: Examining trajectories of COVID-19 disruption over time

Extensive evidence demonstrates that numerous aspects of adolescents’ lives have been disrupted by the COVID-19 pandemic (Angela et al., 2022; Samji et al., 2022). Systematic reviews and meta-analyses have revealed that, since the pandemic’s onset in 2020, adolescent mental (Angela et al., 2022; Kauhanen et al., 2022; Manchia et al., 2022; Samji et al., 2022; Skinner et al., 2021) and physical (Neville et al., 2022) health has deteriorated, adolescents have sustained extensive learning loss in schools (Betthäuser et al., 2022; Hammerstein et al., 2021; König & Frey, 2022), and adolescents have felt isolated and lonely (Angela et al., 2022; Samji et al., 2022). However, because most existing literature is cross-sectional and monocultural, it is unclear if adolescents’ lives have been more disrupted at some points over the course of the pandemic than others. It may be that, as adolescents develop new routines and methods of coping with pandemic life, they experience less disruption in their lives as the pandemic wears on. Alternatively, it may be that as stressors and traumatic experiences accumulate over the course of the pandemic, they overload adolescents’ coping capacities, making the pandemic more disruptive as it wears on (Slomski, 2021). Furthermore, it is unclear if trajectories of adolescent disruption due to COVID-19 are similar or different in different cultural contexts. Given that case rates rose in different places at different times under different sets of restrictions, it is especially important to understand cultural variations in trajectories over time (Bornstein, 2019; Kauhanen et al., 2022; Manchia et al., 2022). In the current study, we answer each of these questions by estimating trajectories of adolescent COVID-19 life disruption over five time points from 2020 to 2022 and examining differences in those trajectories across 12 cultural groups. In so doing, this study unpacks differences in COVID-19 life disruption trajectories attributable to changes in a variety of rapidly changing COVID-19-related variables (e.g., death rates, mitigation strategies, etc.) over the course of a significant period of the pandemic.

## Moving from examining single risk factors to constellations of risk factors: Finding the most important predictors of trajectories of adolescent COVID-19 life disruption

A dizzying array of risk factors has been identified that predict COVID-19 disruption for adolescents (Samji et al., 2022). However, because most studies of COVID-19 disruption only examine one or two risk factors at a time, we do not know which risk factors are actually important in predicting life disruption once several risk factors in adolescents’ ecological context are examined at the same time. Therefore, scientists have called for a move toward examining constellations of risk factors in adolescents’ environments to better understand disruptions related to COVID-19 (Angela et al., 2022; Kauhanen et al., 2022; Manchia et al., 2022; Samji et al., 2022). To examine these constellations of risk factors, the current study is inspired by Bronfenbrenner’s (1977) Social Ecological framework, which conceptualizes individual development as being shaped by individuals’ interactions with the systems in which they are embedded (Angela et al., 2022), and by other COVID-19 researchers’ attempts to group risk factors into personal and cultural contexts (Manchia et al., 2022; de Figueiredo et al., 2021). Specifically, combining these perspectives, we identify and examine three ecological contexts that might affect adolescents’ COVID-19 life disruption: (1) COVID-19’s effects on the societies in which adolescents live, (2) beliefs about COVID-19 and experiences of COVID-19 that adolescents have had in their immediate environment, and (3) adolescents’ behavior problems during the COVID-19 pandemic. We review existing literature examining each of these three ecological contexts, and the current study’s unique contribution to these literatures, in the three subsections that follow.

### Societal-level COVID-19 effects

Several societal-level COVID-19 effects could influence how disruptive the COVID-19 pandemic has been for adolescents. First, how hard COVID-19 hit the society in which the adolescent lives may influence how disruptive the COVID-19 pandemic has been for adolescents. For instance, several systematic reviews and epidemiological datasets have found that mental health problems rise and peak as COVID-19 caseloads increase in a society (Penninx et al., 2022). It may be that as adolescents observe and learn about the scale of death and disease due to COVID-19 in their country, the fear, stress, and trauma associated with such loss leads to greater disruption in their own lives. Second, how stringently a society enforced COVID-19 mitigation measures (e.g., school closures, lockdowns, general societal responses, etc.) may influence how disruptive the COVID-19 pandemic has been for adolescents (Manchia et al., 2022; Samji et al., 2022). Interestingly the direction of these influences is unclear in existing literature, with some studies indicating that more stringent COVID-19 mitigation measures in a society led to less disruption for adolescents (e.g., experiences of fewer negative feelings, greater positive feelings, and fewer mental health issues; Lee et al., 2021, Samji et al., 2022), whereas other studies indicate more stringent COVID-19 measures in a society lead to greater adolescent disruption (Manchia et al., 2022; Samji et al., 2022). In the current study, we examine COVID-19 death rates and measures of how stringent COVID-19 mitigation strategies were as societal-level risk factors that might predict trajectories of adolescent life disruption due to COVID-19.

### Adolescents’ COVID-19 beliefs and experiences

The beliefs adolescents hold about COVID-19 and experiences adolescents have related to COVID-19 in their immediate environment constitute another set of risk factors that may predict how disruptive COVID-19 has been for adolescents. For example, greater confidence in their local government and community’s response to COVID-19 has been associated with better adolescent health (Samji et al., 2022) and greater vaccine uptake (Lansford et al., 2022). Yet, somewhat paradoxically, greater adolescent compliance with COVID-19 pandemic control measures is also associated with more negative adolescent emotions and greater adolescent mental health deterioration (presumably due to social isolation; Samji et al., 2022). Moreover, the trauma of losing someone they knew due to COVID-19 has been linked to tremendous disruption in adolescents’ lives (Hillis et al., 2021; Slomski, 2021), whereas hope and optimism about a COVID-free future has been associated with enhanced adolescent quality of life during the pandemic (Samji et al., 2022). In the current study, we examine how all four of these adolescent COVID-19 beliefs and experiences (confidence in government response to COVID-19, compliance with COVD-19 control measures, losing someone they knew due to COVID-19, hope about COVID-19 ending) predict trajectories of adolescent life disruption during the COVID-19 pandemic.

### Adolescents’ behavior problems

The surge in mental health difficulties and behavior difficulties experienced by adolescents during the COVID-19 pandemic is highly likely to disrupt the lives of adolescents (Angela et al., 2022; Kauhanen et al., 2022; Manchia et al., 2022; Samji et al., 2022). Indeed, internalizing behavioral problems (e.g., sadness, anxiety), problematic alcohol use (Sohi et al., 2022; Yeo et al., 2022), cigarette sales (Asare et al., 2022), and, to a lesser extent, externalizing problems (e.g., anger and getting into arguments; Samji et al., 2022) each increased over the course of the COVID-19 pandemic, and each is associated with greater youth life disruption and impairment (Angela et al., 2022; Kauhanen et al., 2022; Manchia et al., 2022; Samji et al., 2022; Sohi et al., 2022). In the current study we examine associations between all four of these indicators of adolescent behavior problems (internalizing problems, externalizing problems, alcohol use, and smoking) and trajectories of adolescent life disruption during the COVID-19 pandemic.

## Moving from aggregation to disaggregation: Predicting *who* among adolescents experience COVID-19 life disruption and *when* throughout the pandemic such life disruption occurs

Because most studies of risk factors for COVID-19 disruption occur at a single time point, they are unable to disaggregate *who* among adolescents experience COVID-19 disruptions (between-person effects) and *when* such disruptions may occur (within-person effects). However, disaggregating *who* and *when* effects is essential for two reasons.

First, disaggregating these effects ensures that developmental scientists avoid the ecological fallacy (Fisher et al., 2018). The ecological fallacy occurs when effects at the “who” level (i.e., between-person differences in circumstances or attributes) are used to make inferences at the “when” level (within-person, daily/monthly/yearly changes in circumstances or psychological attributes over time; Fisher et al., 2018). A classic example of this fallacy is the exercise-heart attack association. Individuals *who* exercise more are less likely to have heart attacks, but for any given individual, *when* someone exercises more on a specific day they are more likely to have a heart attack (Bauer & Curran, 2013). The ecological fallacy threatens our current knowledge of best practices in medicine, making it essential to disaggregate who and when effects to combat this threat (Fisher et al., 2018). For instance, adolescents *who* comply with COVID-19 regulations more regularly might report less pandemic-related disruption overall because it is more likely that they and their family are kept safe from COVID-19 infection. However, *when* adolescents more regularly comply with COVID-19 regulations than they usually would, their lives might be more disrupted by COVID-19 because such increases in regulation might throw off their normal routines (Samji et al., 2022).

Second, disaggregating these “who” and “when” effects is important because it ensures that associations between risk factors and life disruption are not confounded by fixed between-person differences (i.e., “who” individual characteristics; Rothenberg et al., 2022). For instance, it may be that, adolescents *who* complain more about their life disruption during the pandemic also complain more about their mental health. However, these associations can be examined even more robustly in within-person *when* analyses that use adolescents as their own controls (Rothenberg et al., 2022). These within-person analyses do so by examining how changes in risk factors *above or below an adolescent*’*s average level of those risk factors* contributes to changes in adolescent COVID-19 life disruption. They allow inferences like the following to be made: Even after controlling for whether an adolescent is someone *who* experienced better or worse mental health over the pandemic, compared to their peers, *when* any given adolescent experienced worse mental health than they typically do, their life was also more disrupted by COVID-19.

In sum, disaggregating *who* and *when* effects strengthen the inferences developmental scientists can make about the effects of the COVID-19 pandemic on adolescents by combating the ecological fallacy and controlling for fixed individual characteristics (Fisher et al., 2018; Rothenberg et al., 2022). Knowing *who* among adolescents is most at-risk for pandemic-related life disruption allows policymakers and interventionists to tailor assistance to those who might need it most. Knowing *when* over the course of a pandemic adolescents experience especially high levels of life disruption allows policymakers and interventionists to know when deploying assistance would be most efficacious.

## The current study

The current study examines trajectories of pandemic-related life disruption in 1,080 adolescents from 12 cultural groups in 9 nations. In doing so the current study has three objectives. First, it endeavors to model the best-fitting trajectory that captures how COVID-19 has disrupted adolescents’ lives over the course of the pandemic and see if that trajectory differs by culture. Second, it endeavors to capture how that trajectory is predicted by risk factors related to societal-level COVID-19 effects, adolescents’ beliefs and experiences related to COVID-19, and adolescent behavior problems. Third, by modeling these associations, it endeavors to understand both who among adolescents experience COVID-19 life disruption and when over the course of the pandemic such disruption is especially high.

## Method

### Participants

Participants (Table 1) were drawn from a longitudinal study of parenting and child development and included 1,082 adolescents (*M* = 19.98 years, *SD* = 1.23, 52% girls) from 12 distinct ethnic/cultural groups across nine countries including: Chongqing, China (*n* = 110); Medellín, Colombia (*n* = 80); Naples (*n* = 82) and Rome (*n* = 105), Italy; Zarqa, Jordan (*n* = 100); Kisumu, Kenya (*n* = 88); Manila, Philippines (*n* = 86); Trollhättan/Vänersborg, Sweden (*n* = 88); Chiang Mai, Thailand (*n* = 91); and Durham, NC, United States (*n* = 90 White, *n* = 86 Black, *n* = 76 Latino). Participants were initially recruited into the original study through school letters and continued to participate during the COVID-19 pandemic. Most parents lived together (82%) and were biological parents (97%); nonresidential and non-biological parents also provided data. Sampling included adolescents from each country’s majority ethnic group, except in Kenya where we sampled Luo (third largest ethnic group, 13% of population), and in the U.S., where we sampled equal proportions of White, Black, and Latino families. Socioeconomic status was sampled in proportions representative of each recruitment area. Child age and gender did not vary across countries. Data for the present study were drawn from interviews at five time periods during the first 2.5 years of the COVID pandemic between March 2020 and July 2022.

### Procedures

At the beginning of the study, all adolescents provided consent to participate and all procedures were approved by the Duke University Review board as well as review boards at each local institution. All interviews with adolescents were conducted exclusively online, by postal mail, or by telephone because of COVID-19-related health concerns, and typically lasted 5 min or less. Forward and backward translation of items ensured linguistic and conceptual equivalence of measures (Erkut, 2010; Peña, 2007). Interviews with adolescents began on March 11, 2020 (the first week of COVID-19-induced lockdowns in most of the countries) and continued to July 31, 2022.

Data collection spanned different amounts of time to collect each wave in each nation due to COVID-19 pandemic restrictions. Therefore, aligning with best practices in longitudinal literature (Bauer & Curran, 2013), we “turned” the data to examine trajectories of adolescent disruption over time, instead of by “wave” of data collection in each culture. Specifically, we examined trajectories of adolescent disruption over five time periods, each coinciding with a half year of the pandemic: (1) March 2020–September 2020, (2) September 2020–March 2021, (3) March 2021–September 2021, (4) September 2021–March 2022, and (5) March 2022–July 2022. Given this data collection time frame varied across sites, data were missing for some adolescents at some time points. On average, adolescents completed 2.84 reports of COVID-19 related life disruption. Only 46 adolescents (4.25%) were missing reports of COVID-19 related disruption at all time points. Adolescents with missing reports did not differ from those with complete reports on age (*t*(877) = 0.50, *p* = .61), or parent education (*t*(944) = 0.67, *p* = .51), but were more likely to be boys (χ^2^ [1] = 17.67, *p* < .01). To account for any systematic differences in predictions based on missingness, all three of these covariates were controlled in subsequent analyses.

### Measures

#### Covariates

In all analyses where we examined risk factors that predicted trajectories of COVID-19 disruption, we controlled for adolescent age (in years), adolescent gender (0 = female, 1 = male), and number of years of parent education.

#### Culture group membership

Cultural group membership was captured via a categorical variable that identified each of the 12 cultural groups in the current study.

#### Societal-level COVID-19 effects

We measured societal-level COVID-19 effects by examining COVID-19 death rates in each country over time, and how stringent COVID-19 mitigation strategies were in each nation over time.

##### COVID-19 death rates.

We calculated average COVID-19 death rates per 100,000 people in each of the nine nations during each of the five time periods examined in the current study. Average death rates were calculated from data provided by Our World in Data (Mathieu et al., 2020), the same data source used by Google, the New York Times, and many other organizations.

##### Stringency of COVID-19 mitigation strategies.

How stringently a nation implemented COVID-19 mitigation strategies was measured in each of the nine nations during each of the five time periods examined in the current study via the COVID-19 Containment and Health Index (Hale et al., 2021; Mathieu et al., 2020). This Index measures the strictness of government policies related to COVID-19 on a 0–100 scale (with 100 being the most strict) and is generated based on 13 areas of COVID-19 policy: school closures; workplace closures; cancellation of public events; restrictions on public gatherings; closures of public transport; stay-at-home requirements; public information campaigns; restrictions on internal movements; international travel controls; testing policy; extent of contact tracing; face coverings; and vaccine policy (Mathieu et al., 2020). Since the beginning of the pandemic, this measure has been calculated in each country every day by researchers at Our World in Data, so we were able to calculate average scores on this Index in each country during each time period examined in the current study.

#### Experiences related to COVID-19 scale

All measures of adolescents’ COVID-19 beliefs and experiences, behavior problems, and COVID-19 related disruption examined in the current study emerge from adolescent reports on the *Experiences Related to COVID-19* questionnaire (Skinner et al., 2021; see Supplemental Materials EC19-R for a copy of the measure) that has been used by numerous investigators world-wide, including in the cultural groups examined here (e.g., Davidson et al., 2021; Lansford et al., 2022). This self-report questionnaire asks participants various questions about their experiences related to COVID-19.

#### Adolescents’ COVID-19 beliefs and experiences

We measured four aspects of Adolescents’ COVID-19 beliefs and experiences during each of the five study time periods: adolescent confidence in government response to COVID-19, compliance with COVID-19 control measures, death of someone they knew due to COVID-19, and hope that the COVID-19 pandemic would end.

##### Confidence in government response to COVID-19.

Adolescents rated the following statement on a 1 = *Strongly Disagree* to 4 = *Strongly Agree* scale: “I am confident the government is handling the COVID-19 response in the best possible manner.” Notably, adolescents were asked to take into account local, state/regional, and national government responses as they responded to this statement.

##### Compliance with COVID-19 control measures.

Adolescents rated the following statement on a 1 = *Strongly Disagree* to 4 = *Strongly Agree* scale: “I complied with the rules and suggestions of the government and healthcare system to try to contain the virus.”

##### Death of someone they knew due to COVID-19.

Adolescents answered 0 = *No* or 1 = *Yes* to the following question: “Has someone close to you lost their life due to COVID-19.”

##### Hope of COVID-19 pandemic ending.

Adolescents rated the following statement on a 1 = *Strongly Disagree* to 4 = *Strongly Agree* scale: “I am hopeful that the COVID-19 virus will resolve over time and I have a good outlook toward the future.”

#### Adolescents’ behavior problems

We measured four indicators of adolescents’ behavior problems during the COVID-19 pandemic during each of the five study time periods: internalizing problems, externalizing problems, alcohol use, and smoking. For each behavioral problem indicator, adolescents answered the following prompt on a 1 = *Made it a lot better* to 5 = *Made it a lot worse* scale: “Thinking about your life right now, how has the COVID-19 pandemic affected each of the following ... ” All measures were standardized with a mean of 0 and standard deviation of 1.

##### Internalizing problems.

Aligning with existing work (Skinner et al., 2021, 2022), internalizing problems were measured by averaging the “Your feelings of anxiety” and “Your feelings of depression/sadness” items.

##### Externalizing problems.

Aligning with existing work (Skinner et al., 2021), externalizing problems were measured by averaging the “Your anger” and “Your getting into arguments” items.

##### Alcohol use.

Alcohol use was measured via the score on the “Your alcohol use” item.

##### Smoking.

Smoking was measured via the score on the “Your smoking” item.

#### Adolescents’ COVID-19-related life disruption

Aligning with existing work (Skinner et al., 2021), adolescents rated the extent to which COVID-19 had disrupted their life during each of the five study time periods by rating the following question from 1 = *Not at all disruptive* to 10 = *Extremely Disruptive*: “Please rate how much the COVID-19 outbreak has been disruptive to you personally. Think about your daily routines, work, and family life.”

### Analytic plan

Following expert recommendations (Bauer & Curran, 2013), we estimated a series of multilevel models in SAS 9.4 using restricted maximum likelihood estimation to examine all study objectives. To examine the first study objective, we identified the best-fitting trajectory for modeling adolescent life disruption due to COVID-19 across the entire sample and examined whether that trajectory varied in different cultural contexts, using chi-square tests to compare alternative models (Bauer & Curran, 2013). To model trajectories, our five time periods were coded such that time period 0 = March 2020–September 2020, time period 1 = September 2020–March 2021, time period 2 = March 2021–September 2021, time period 3 = September 2021–March 2022, and time period 4 = March 2022–July 2022. Then, to examine our second study objective, we group-mean centered each risk factor and used them to predict the trajectory of adolescent life disruption due to COVID-19 after controlling for effects of adolescent age and gender, parent education, and cultural group membership. This analysis provides insight into *who* among adolescents experienced COVID-19 life disruption over the course of the pandemic (Bauer & Curran, 2013). Finally, to examine our third study objective, we person-mean centered each risk factor and used these person-mean centered risk factors to predict deviations from the overall trajectory of adolescent life disruption due to COVID-19. This analysis provides insight into *when* during the pandemic experiencing especially high levels of a risk factor (compared to what an adolescent normally faces) leads to especially high levels of adolescent COVID-19 life disruption (Bauer & Curran, 2013).

We used interaction terms to examine how both the “who” and “when” associations of risk factors with adolescent COVID-19 life disruption changed over the course of the pandemic. In both instances, risk factors were examined in risk factor * time interaction terms to examine linear changes in risk factors’ associations with adolescent COVID-19 life disruption over the course of the pandemic, and risk factors were examined in risk factor * time^2^ interaction terms to determine quadratic changes in risk factors’ associations over the course of the pandemic. In instances where these interaction terms were not statistically significant, they were pruned from the final model to ensure appropriate interpretability of risk factor main effects. In instances where “who” risk factor * time interactions were significant, SAS 9.4 “proc estimate” statements were used to examine how intercepts and slopes changed in adolescents who experienced 1 standard deviation below average, average, and 1 standard deviation above average levels of a risk factor. In instances where “when” risk factor * time interactions were significant, SAS 9.4 “proc estimate” statements were used to examine whether the association between a “when” risk factor and adolescent COVID-19 related life disruption was significant in each of the five half-year intervals since the pandemic began. If the estimate of this association was significant in a particular half-year interval, that estimate was reported.

Importantly, all models that examined the effects of risk factors on adolescent life disruption due to COVID-19 controlled for adolescent gender and age, number of years of parent education (to adjust estimates for family socioeconomic status), and adolescent cultural group membership (to adjust estimates for other potential differences due to country of origin or, in the case of the United States, race/ethnicity, because US Black, Latino, and White groups were each coded as separate cultural groups). We controlled for these covariates because they have emerged as powerful predictors of COVID-19 case rates and other health care disparities over the course of the pandemic and beyond (e.g., Yancy, 2020).

### Measure limitations

Before describing study results, several limitations of study measures should be acknowledged. Specifically, most of the risk factors examined in this study are reported by a single reporter and/or measured by a single item. Collection of data in this way was necessary at the height of the COVID-19 pandemic, to ensure that data collection could occur rapidly to capture adolescent functioning in real time, while also not burdening participants dealing with tremendous amounts of stress and trauma because of the pandemic (Skinner et al., 2021). Study authors are confident these measures are reliable and valid but felt that these limitations should be acknowledged in the interest of full transparency. We desired to provide context to readers who might be examining this research many years after the height of the COVID-19 pandemic and wonder about our design choices.

## Results

All study descriptive statistics can be found in Table 2, and zero-order correlations between all study variables can be found in Table 3. Notably, Table 3 indicates that all risk factors except for alcohol use were correlated with adolescent COVID-19-related life disruption. Higher COVID-19 death rates, greater compliance rates with COVID-19 measures, stringency of COVID-19 mitigation strategies, knowing someone who died due to COVID-19, internalizing problems, externalizing problems, and smoking were all associated with greater overall adolescent-reported COVID-19-related life disruption. More stringent government mitigation strategies, greater confidence in government response to COVID-19, and greater hope that the COVID-19 pandemic would end was associated with less overall adolescent-reported COVID-19 related life disruption.

Preliminary multilevel models with no predictors revealed that between-person “who” effects accounted for 40.35% (*p* < .01) of variance in adolescent-reported COVID-19 life disruption scores, compared to 59.65% (*p* < .01) of variance accounted for by within-person “when” effects (i.e., Intraclass Correlation Coefficient = .4035). The significant amount of variation in adolescent-COVID-19 life disruption scores at the within-person “when” level supports our strategy to examine risk factors that might predict both *who* among adolescents experiences COVID-19 life disruption and *when* adolescents might experience such disruption.

### Objective 1: Examining trajectories of adolescent COVID-19-related life disruption

A quadratic trajectory best characterized how adolescents perceived that COVID-19 disrupted their life over the course of the pandemic, as it fit the data better than a linear trajectory (χ^2^(4) = 26.8, *p* < .01). A cubic trajectory did not fit the data significantly better than the quadratic trajectory (χ^2^(4) = 4.9, *p* = .29). Moreover, this quadratic trajectory fit the data best if random linear and quadratic effects were estimated, and if the residual error structure was heteroscedastic (χ^2^(4) = 19.8, *p* < .01).

**Figure 1.**
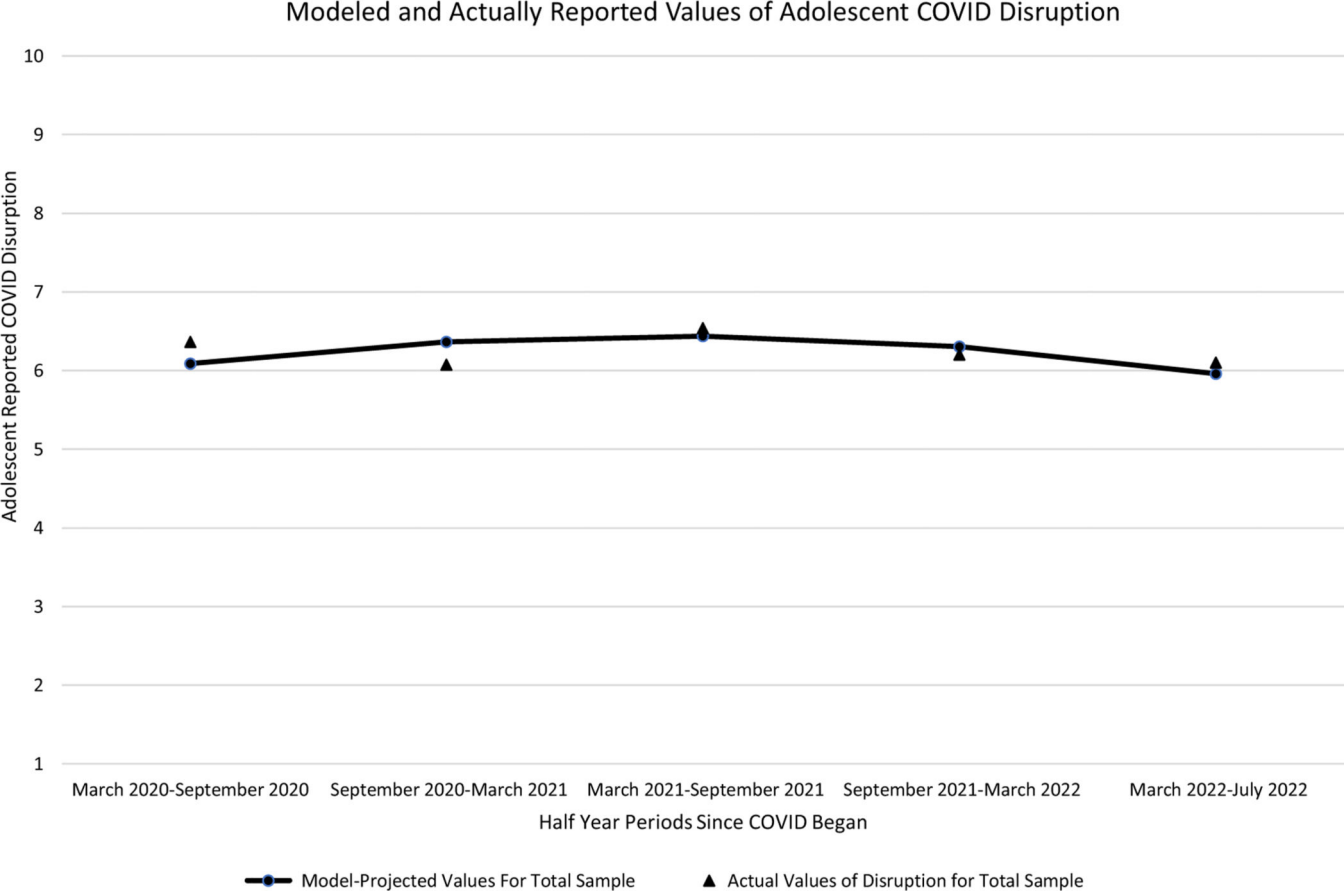
Modeled trajectory of adolescent-reported COVID-19 life disruption compared to actual mean levels of adolescent-reported COVID-19 life disruption in current sample. *Note.* Trajectory is a quadratic growth curve trajectory modeled in a multilevel modeling framework (see results for further details).

This quadratic trajectory revealed that in the first 6 months of COVID, adolescents reported an average score of 6.09 out of 10 on the COVID-19 life disruption scale, and this score increased linearly by 0.38 points for each additional half year (Table 4). However, this linear increase itself slowed over time at a rate of 0.10 points each half year (Table 4). This trajectory in its entirety is depicted in Figure 1, where average COVID-19 life disruption scores increased over time to a high of approximately 6.5 from March 2021 to September 2021, before subsequently decreasing over time back to a score of approximately 6 in March 2022–July 2022.

#### Differences by culture

**Figure 2.**
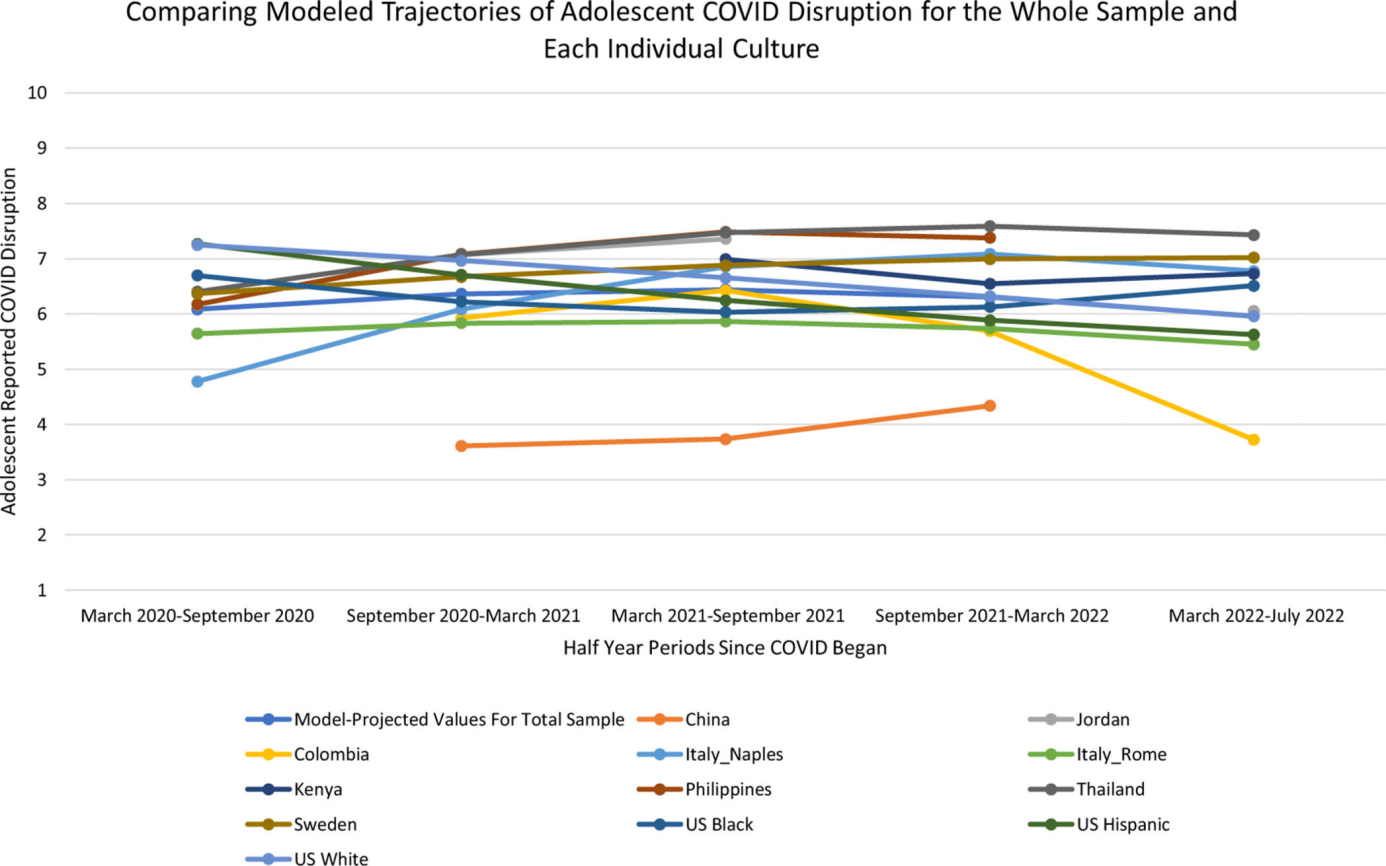
Differences in modeled trajectories of adolescent-reported COVID-19 life disruption across cultures. *Note.* Trajectories in each culture are only modeled at time points where the adolescents in that culture reported on their life disruption due to COVID-19. So for instance, the trajectory for the Chinese sample consists of only three time points because Chinese adolescents only reported on their COVID-19-related disruption between September 2020 and November 2021.

Next, we examined whether this trajectory of adolescent-reported COVID-19 life disruption scores differed by cultural context. Findings reported in Table 4 and Figure 2 indicate significant differences across cultures. All significant differences reported below are in relation to the average trajectory of adolescent disruption seen across all cultures. At the beginning of the pandemic (i.e., at “intercept”), adolescents from four cultural groups: (Chonqing, China; Medellin, Colombia; Naples, Italy; and Rome, Italy) reported significantly lower COVID-19 life disruption scores than adolescents in other cultural groups, and adolescents from two cultural groups (US White and Latino adolescents) reported significantly higher COVID-19 life disruption scores than adolescents in other cultural groups (Table 4). Additionally, over the course of the pandemic, adolescents in three cultural groups (Medellin, Colombia; Naples, Italy; and Manila, Philippines) reported linear increases in COVID-19 life disruption that were higher than the overall sample average, and adolescents in three cultural groups (US Black; US White; and US Latino) reported linear increases in COVID-19 life disruption that were lower than the overall sample average (Table 4). Finally, as indicated by significant quadratic effects, over the course of the pandemic adolescents in one cultural group (Medellin, Colombia) reported more rapid deceleration in linear increases of COVID-19 life disruption than the overall sample average, and adolescents in one cultural group (US Black) reported more rapid acceleration in linear increases of COVID-19 life disruption than the overall sample average (Table 4).

Collectively, these findings are depicted in Figure 2 which demonstrates significant heterogeneity in adolescent COVID-19 life disruption starting points and rates of change by culture. Adolescents from most cultures at most time points reported their life had been disrupted by COVID-19 between a “6” or a “7” on a 1–10 scale across the first 2.5 years of the pandemic, with peak levels of disruption occurring between March 2021 and March 2022. However, some cultural groups demonstrated different patterns of COVID-19 disruption. The COVID-19 pandemic was most disruptive to the lives of US White and US Latino adolescents at its onset (with reported disruption scores over 7), before steadily decreasing over time. Adolescents from Rome, Italy and Chongqing, China reported low levels of disruption throughout the pandemic (as their scores remained below “6”). The pandemic was most disruptive to the lives of adolescents from Thailand and the Philippines from September 2021 to July 2022, during which adolescents from both cultures reported disruption scores well above “7.” Finally, the disruptive effects of the pandemic for adolescents from Colombia seemed to decrease dramatically from March 2022 to July 2022, as these adolescents’ disruption scores fell below “4.” The next analyses attempt to understand the ecological contextual reasons for differences seen in trajectories across cultures.

### *Objectives 2 & 3: Examining risk factors’ association with* who *among adolescents experience COVID-19 life disruption and* when *during the pandemic such disruption occurs*

We describe results by ecological context below. Given the plethora of significant findings, we describe broad patterns of findings. Specific empirical results can be found in Table 5.

#### Societal-level COVID effects

##### National COVID-19 death rates.

**Figure 3.**
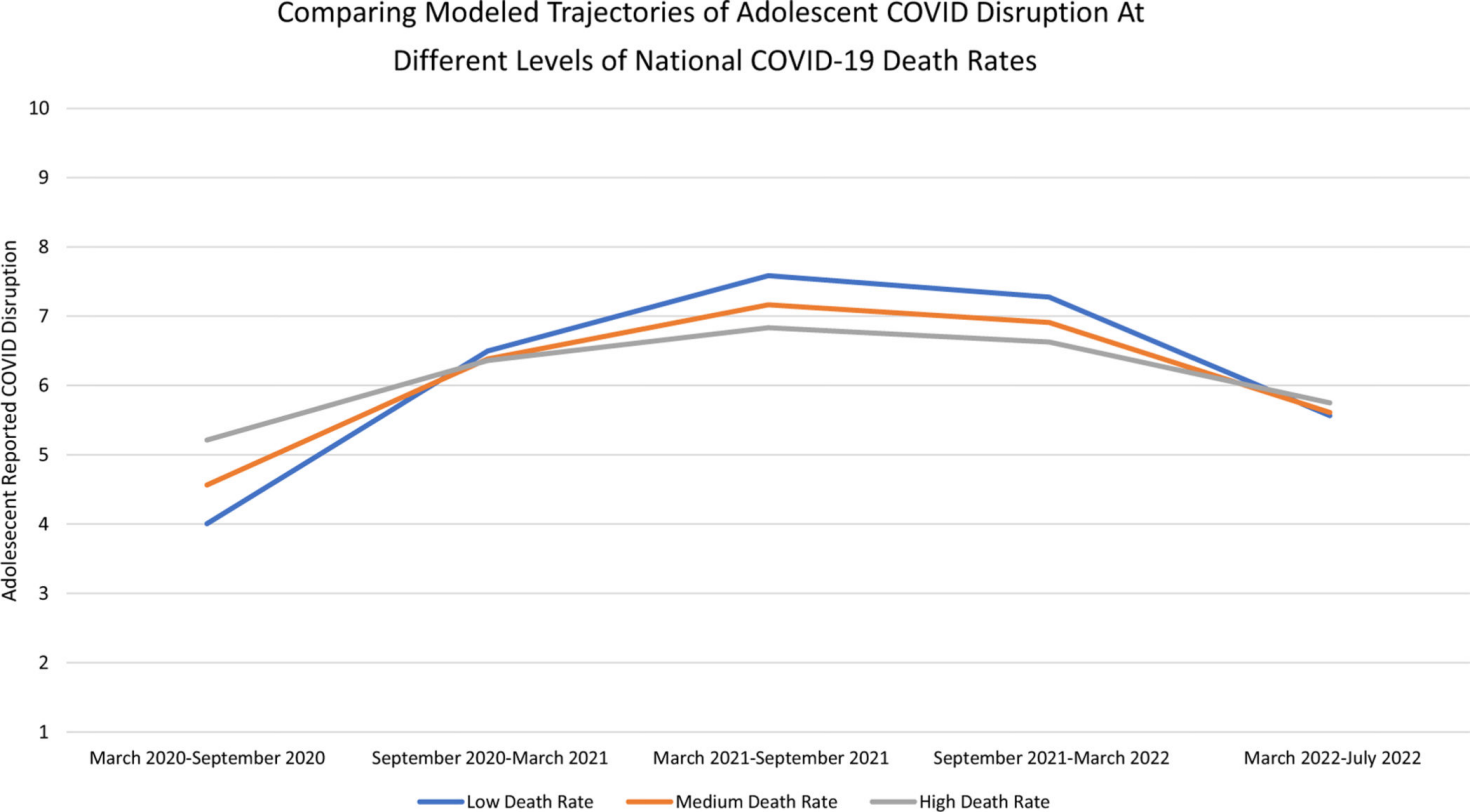
Differences in modeled trajectories of adolescent-reported COVID-19 life disruption at different levels of national COVID-19 death rates. *Note.* Low Death Rate indicates adolescents who lived in nations that scored one standard deviation below average on COVID-19 death rate, Medium Death Rate indicates adolescents who lived in nations with average COVID-19 death rates, and High Death Rate indicates adolescents who lived in nation that scored one standard deviation above average on COVID-19 death rate.

National COVID-19 death rates were significant predictors of intercept, linear slope, and quadratic slope of adolescent-reported COVID-19 life disruption. When probed, these combined effects indicated that adolescents *who* were from countries with higher overall death rates compared to other countries in the sample reported greater life disruption during COVID-19’s first 6 months, until September 2020 (see Figure 3, which compares trajectories of Adolescent COVID-19 Disruption in nations with one standard deviation below average, average, and one standard deviation above-average COVID-19 death rates in the sample). Then, the pattern switched, and from September 2020 to March 2022, adolescents *who* lived in countries with lower overall death rates compared to other countries in the sample reported greater life disruption due to the COVID-19 pandemic (Figure 3). Finally, from March 2022 to July 2022, it appears that national COVID-19 death rates were not associated with *who* among adolescents experienced disruption due to COVID-19.

National COVID-19 related death rates were also a significant predictor of *when* during the pandemic adolescents experienced life disruption (Table 5). From March 2022 to July 2022, *when* adolescents lived in nations that experienced death rates that were higher than typical for that nation, adolescents experienced greater COVID-19 life disruption (*B* = .05, *p* < .01).

##### Stringency of COVID-19 mitigation strategies.

Stringency of COVID-19 mitigation strategies was a significant predictor of the intercept, but not linear or quadratic slope, of adolescent-reported COVID-19 life disruption. Therefore, adolescents *who* were from countries with less stringent COVID-19 reduction measures compared to other countries reported greater COVID-19 disruption at the beginning of the pandemic, and this association persisted throughout the COVID-19 pandemic (Table 5).

Stringency of COVID-19 mitigation strategies was also a significant predictor of *when* during the pandemic adolescents experienced life disruption (Table 5). Specifically, for the first 6 months of the COVID-19 pandemic (March 2020–September 2020) *when* adolescents lived in nations that enacted COVID reduction measures that were less stringent than typical for that nation, adolescents reported greater COVID-19 disruption (*B* = −.13, *p* < .01). This effect dissipated after the first 6 months of the pandemic.

#### Adolescents’ COVID-19 beliefs and experiences

##### Confidence in government response to COVID-19.

Confidence in Government Response to COVID-19 was a significant predictor of the intercept, but not linear or quadratic slope, of adolescent COVID-19 life disruption (Table 5). Therefore, adolescents *who* reported less confidence in their government’s response to COVID-19 compared to their peers reported greater COVID-19 disruption at the beginning of the pandemic, and this association persisted across the entirety of the pandemic.

Confidence in government response to COVID-19 was also a significant predictor of *when* during the pandemic adolescents experienced life disruption (Table 5). Specifically, for the first 6 months of the COVID-19 pandemic (March 2020–September 2020) *when* adolescents were less confident in their government’s response than was typical for them, adolescents reported greater COVID-19 life disruption (*B* = .27, *p* < .01). This effect also dissipated after the first 6 months of the pandemic.

##### Compliance with COVID-19 control measures.

**Figure 4.**
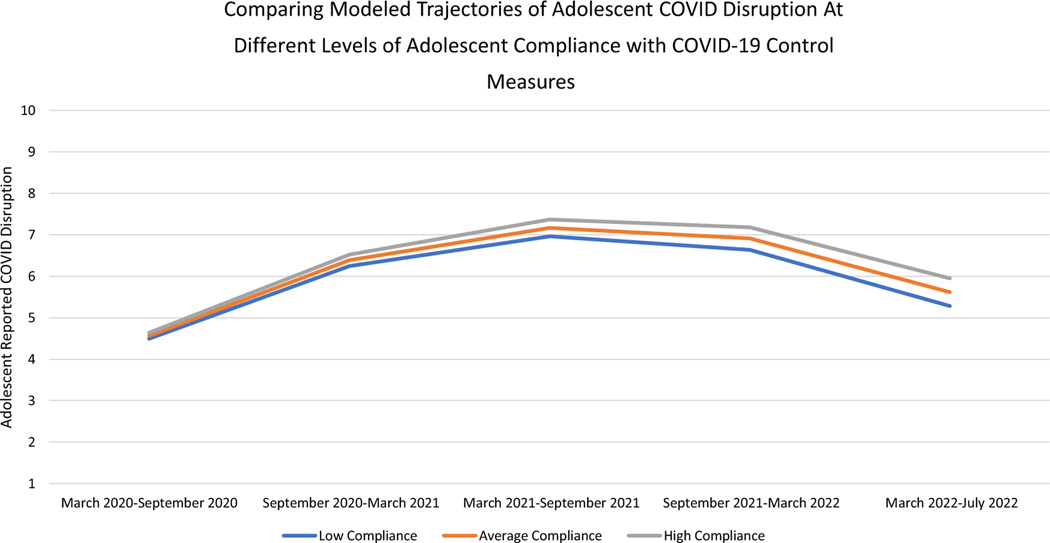
Differences in modeled trajectories of adolescent-reported COVID-19 life disruption at different levels of adolescent compliance with COVID-19 control measures. *Note.* Low Compliance indicates adolescents who scored one standard deviation below average on compliance, Medium Compliance indicates adolescents who had average compliance scores, and High Compliance indicates adolescents who scored one standard deviation above average on compliance.

Additionally, compliance with COVID-19 control measures was a significant predictor of the linear slope, but not the intercept or quadratic slope, of adolescent COVID-19 life disruption trajectories (Table 5). Adolescents *who* reported greater compliance with COVID-19 control measures compared to their peers did not report greater COVID-19 disruption at the beginning of the pandemic but did report greater COVID-19 life disruption as the pandemic wore on after September 2020 (see Figure 4, which compares COVID-19 life disruption in adolescents with 1 standard deviation below average, average, and 1 standard deviation above average levels of compliance).

Moreover, across the entire first 2.5 years of the pandemic, at times *when* any given adolescent reported greater compliance with COVID-19 control measures than was typical for that adolescent, they also reported more COVID-19 life disruption (Table 5).

##### Death of someone they knew due to COVID-19.

Adolescents *who* reported someone close to them losing their life during COVID-19 reported greater COVID-19 life disruption, compared to those who did not have such an experience (Table 5).

##### Hope of COVID-19 pandemic ending.

Adolescents’ hopes for the end of the COVID-19 pandemic did not predict *who* among adolescents or *when* during the pandemic adolescents experienced COVID-19 life disruption (Table 5).

#### Adolescents’ behavior problems

##### Internalizing problems.

Internalizing problems significantly predicted the intercept, but not the linear or quadratic slope, of adolescent-reported COVID-19 life disruption trajectories. Adolescents *who* experienced more internalizing problems compared to their peers reported greater COVID-19 life disruption at the beginning of the pandemic, and this association persisted throughout the pandemic (Table 5). Moreover, across the entire first 2.5 years of the pandemic, at times *when* adolescents experienced more internalizing problems than was typical for them, they also experienced greater COVID-19 life disruption (Table 5).

##### Externalizing problems.

Like internalizing problems, externalizing problems significantly predicted the intercept, but not the linear or quadratic slope, of adolescent-reported COVID-19 life disruption trajectories (Table 5). Adolescents *who* experienced more externalizing problems compared to their peers reported greater COVID-19 life disruption at the beginning of the pandemic, and this association persisted throughout the pandemic.

In contrast, *when* an adolescent experienced more externalizing problems than was typical for that adolescent, these more-than-typical problems were not associated with adolescent COVID-19 related life disruption (Table 5).

##### Alcohol use.

Adolescents’ self-reported changes in alcohol use did not predict *who* among adolescents or *when* during the pandemic adolescents experienced COVID-19 life disruption (Table 5).

##### Smoking.

**Figure 5.**
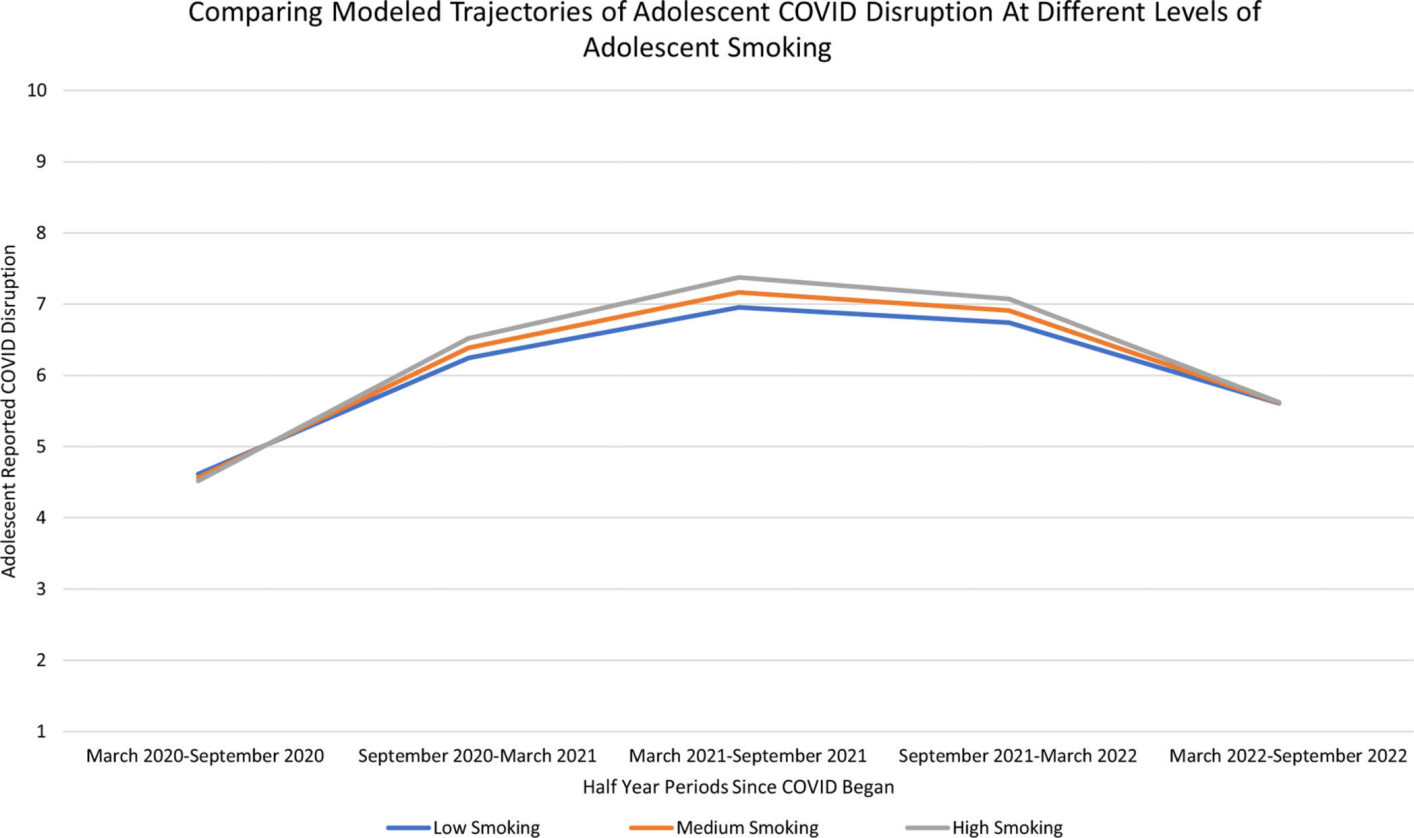
Differences in modeled trajectories of adolescent-reported COVID-19 life disruption at different levels of adolescent smoking. *Note.* Low Smoking indicates adolescents who scored one standard deviation below average on smoking, Medium Smoking indicates adolescents who had average smoking scores, and High Smoking indicates adolescents who scored one standard deviation above average on smoking.

Smoking was a significant predictor of the linear and quadratic slope, but not the intercept, of adolescent COVID-19 life disruption trajectories (Table 5). Adolescents *who* reported greater smoking compared to their peers did not report greater COVID-19 disruption at the beginning of the pandemic or end of the pandemic but did report greater COVID-19 life disruption between September 2020 and March 2022 (see Figure 5 which compares COVID-19 life disruption in adolescents with 1 standard deviation below average, average, and 1 standard deviation above average levels of smoking). Moreover, across the entire first 2.5 years of the pandemic, at times *when* adolescents smoked more than was typical for them, they also experienced greater COVID-19 life disruption (Table 5).

## Discussion

Numerous risk factors identified *who* among adolescents and *when* throughout the pandemic adolescents experienced COVID-19 life disruption. Given that developmental scientists have identified the sustained deleterious impact of COVD-19 life disruption on adolescent daily routines, academic achievement, work, and family and peer relationships, learning more about adolescent trajectories of COVID-19 life disruption and the risk factors that disrupt or exacerbate them is critical to helping adolescents cope with, and recover from, the COVID-19 pandemic (Angela et al., 2022; Kauhanen et al., 2022; Manchia et al., 2022; Samji et al., 2022; Skinner et al., 2021).

### Objective 1: Examining trajectories of adolescent COVID-19-related life disruption

Over the first 2.5 years of the COVID-19 pandemic, it appears that the average level at which adolescents’ lives were disrupted remained relatively stable, as evidenced by adolescents’ average life disruption scores ranging between 6 and 7 on a 10-point scale across all five time points. This trajectory indicates that adolescents in our sample on average viewed their lives as disrupted by COVID-19 and that this disruption did not improve even after most nations in this sample lifted their most strenuous COVID-19 mitigation strategies (such as lockdowns). That adolescents saw their lives as disrupted by COVID-19 is not surprising, given the enormous social, scholastic, and mental health toll COVID-19 has taken on adolescents (Samji et al., 2022). The fact that adolescents still viewed their lives as essentially just as disrupted by COVID-19 in July 2022 as they did at the beginning of the pandemic is more worrying and may evidence the long reach of COVID-19 on deleterious adolescent development (Angela et al., 2022; Kauhanen et al., 2022; Manchia et al., 2022; Samji et al., 2022).

Taking a “glass half full approach,” however, the fact that average adolescent pandemic disruption scores stayed between 6 and 7 indicates that many adolescents developed a sense of resilience over the course of the pandemic and did not find the cumulative effects of the pandemic to drastically increase their subjective sense of life disruption (Slomski, 2021). Thus, the average pandemic trajectory that emerged in this study speaks to both the long-term toll that the pandemic has taken on subjective adolescent well-being and the relative resilience of adolescents in the face of the worst periods of the pandemic.

Of course, the trajectory of adolescents’ reported pandemic life disruption showed some variability, as it increased from an average score of 6.09 in the pandemic’s first 6 months to high score of approximately 6.5 from March 2021 to September 2021 before subsequently decreasing to approximately a score of 6 in data obtained in March 2022–July 2022. These increases and decreases align closely with increases and decreases in case and death rates worldwide due to the COVID-19 pandemic. Indeed, the correlation between cumulative worldwide deaths due to COVID-19 that occurred in each of the five 6-month time intervals examined here and the average COVID-19 life disruption scores reported by adolescents in this sample is *r* = .46 (*p* < .01). Simply put, adolescent COVID-19 disruption scores in our sample rose and fell as worldwide pandemic case and death rates rose and fell.

Finally, it is notable that, although most adolescents scored between “6” and “7” on the COVID-19 life disruption scale across most cultures for most of the pandemic, trajectories of COVID-19 pandemic life disruption did significantly vary across cultural groups. Often, it seems like heterogeneity in trajectories can be explained by heterogeneity in the COVID-19 pandemic’s effects in different cultural groups.

For instance, for US White and Latino adolescents, disruption scores were highest at the beginning of the pandemic and steadily decreased over time (Figure 2). This pattern coincided with school lockdowns in the United States; schools, universities, and workplaces in the United States went completely virtual at the pandemic’s beginning (approximately March 2020–September 2020), before returning in hybrid virtual/in-person manner in the middle of the pandemic (approximately September 2020–September 2021), and then returning to fully in-person in most instances (September 2021–July 2022; Hale et al., 2021; Mathieu et al., 2020). It could be that, as schools and workplaces steadily returned to “normal,” the myriad social and scholastic routines by which adolescents lived their lives also returned to normal, and pandemic-related life disruption decreased for those US White and Latino adolescents. Similarly, the pandemic was most disruptive to the lives of adolescents in Thailand and the Philippines over September 2021–July 2022. These increases coincided with an explosion of COVID-19 cases and deaths in these countries (Mathieu et al., 2020), as well as new school closures in Thailand (Hale et al., 2021) and continued school closures in the Philippines (Hale et al., 2021) during that time frame. In Colombia, adolescent-reported COVID-19 life disruption scores decreased dramatically in March 2022–July 2022, right as COVID deaths and case rates leveled off throughout that nation (Mathieu et al., 2020). In China, adolescent COVID-19 related life disruption was low throughout the pandemic, which coincided with low reported case rates and deaths in China throughout the period September 2020–March 2022 (Mathieu et al., 2020). Unfortunately, no data on adolescent COVID-19 life disruption are available from China since March 2022 after which China reversed its Zero-COVID policy (Hale et al., 2021). The one pattern of cultural heterogeneity that is less explainable is that seen in Rome, Italy. In Rome, adolescents reported among the lowest COVID-19 life disruption scores throughout the pandemic, despite Italy being among the first and hardest-hit nations by COVID-19 and experiencing expansive lockdowns due to COVID-19. It will be interesting to learn if our finding in the Roman context replicates in other empirical investigations of adolescent COVID-19 related life disruption.

### Objective 2: Finding the most important predictors of trajectories of adolescent COVID-19 life disruption

Answering calls from prevention scientists around the world (Angela et al., 2022; Kauhanen et al., 2022; Manchia et al., 2022; Samji et al., 2022; Sohi et al., 2022), we set out to move beyond examining single risk factors at single points in time to understand which risk factors emerge as the most important in predicting adolescent COVID-19 related life disruption over time. Instead of finding that one or two risk factors emerged as the most important indicators of adolescent COVID-19 life disruption, we found that multiple risk factors at multiple ecological levels of development predicted adolescents’ COVID-19 life disruption. Both societal-level variables (National COVID-19 Death Rates, Stringency of COVID-19 Mitigation Strategies) predicted adolescent COVID-19 disruption, as did three of four variables capturing adolescents’ beliefs and experiences (Confidence in Government Response to COVID-19, Compliance with COVID-19 Control Measures, and Death of Someone They Knew Due to COVID-19), and three of four variables capturing adolescents’ behavior problems (Internalizing, Externalizing, and Smoking Problems). In other words, adolescent life disruption due to the COVID-19 pandemic appears less like some sort of viral pathogen attributable to one or two risk factors, and more like a developmental phenomenon that unfolds over time and is best predicted by a constellation of risk factors as adolescents are shaped by their interactions with the systems in which they are embedded (Bronfenbrenner, 1977). This finding is encouraging, because it means that several different COVID-19 recovery interventions launched at multiple ecological levels could improve adolescents’ sense of life disruption due to the COVID-19 pandemic.

It is also notable that the effects of certain risk factors persisted throughout the COVID-19 pandemic, so prioritizing interventions to address these risk factors may be especially powerful in remediating deleterious effects of the pandemic across ontogeny. Adolescents who were from nations with less stringent COVID-19 mitigation strategies, who had less confidence in government response to COVID-19, who experienced the death of a loved one during the pandemic, and who experienced more internalizing and externalizing problems were more likely to experience COVID-19 life disruption across the entirety of the first 2.5 years of the COVID-19 pandemic.

Many interventions might mitigate the effects of persistent deleterious risk factors. Stringent COVID-19 mitigation strategies on a national level are likely to decrease adolescents’ life disruption due to COVID-19, especially if adolescents’ are confident in this government response (Lansford et al., 2022; Samji et al., 2022). Providing support and counseling for the hundreds of thousands of children and adolescents worldwide whose parents passed away during the first 2.5 years of the COVID-19 pandemic has the potential to decrease the extent to which adolescents perceive their lives as totally upended by the pandemic (Slomski, 2021). Finally, screening for, and providing mental health services to reduce, adolescent internalizing and externalizing problems is likely to reduce COVID-19 pandemic-related life disruption in many different cultures (de Figueiredo et al., 2021; Samji et al., 2022).

### *Objective 3: Predicting* who *among adolescents experience covid-19 life disruption and* when *throughout the pandemic such life disruption occurs*

Disaggregating *who* among adolescents experience COVID-19 life disruption and *when* throughout the pandemic such life disruption occurs provides additional insights into how the broad constellation of risk factors in adolescents’ ecological contexts are associated with adolescents’ pandemic-related life disruption. These *who* and *when* insights avoid the ecological fallacy, and account for fixed, individual characteristics (Fisher et al., 2018; Rothenberg et al., 2022). Many of these insights provide empirical evidence that can guide debates raging in popular culture about the effects of COVID-19 on adolescents.

#### Do COVID-19 mitigation strategies make the lives of adolescents better or worse?

For instance, one question that is debated in popular COVID-19 discourse is: Do COVID-19 mitigation strategies (e.g., lockdowns, school closures) make the lives of adolescents better or worse (Hale et al., 2021)? In the current sample, the answer to this question seems to be: It depends on *who* these strategies are applied to and *when* these strategies are measured.

The presence of comprehensive COVID-19 mitigation strategies at a national level seems to make COVID-19 less disruptive in the lives of adolescents. Adolescents *who* were from nations with more stringent COVID-19 mitigation strategies experienced less COVID-19 related life disruption throughout the pandemic compared to their peers. Moreover, for the first 6 months of the pandemic, *when* adolescents lived in nations that enacted more stringent COVID-19 mitigation strategies than those nations did throughout the rest of the pandemic, these adolescents also experienced less COVID-19 life disruption.

However, if one measures individual adolescents’ *compliance* with COVID-19 mitigation strategies, then it appears adolescents *who* reported greater compliance with COVID-19 mitigation strategies than their peers reported more COVID-19 life disruption after the first 6 months of the pandemic (i.e., September 2020). Similarly, at any given time point, *when* adolescents reported greater compliance with COVID-19 mitigation strategies than was typical for them, they also reported more COVID-19 life disruption.

Collectively, these findings appear to indicate that national COVID-19 mitigation strategies implemented throughout the pandemic protect against adolescent life disruption, but as the pandemic wears on, compliance with such measures leads adolescents to perceive greater life disruption. Disaggregating *who* and *when* effects underscores that life-saving COVID-19 mitigation strategies are important to adolescent well-being, but that it might also be worthwhile to understand exactly when adolescent compliance with those strategies disrupts adolescents’ lives. Doing so would allow interventionists to discern whether there are ways to minimize adolescent life disruption in the midst of implementing COVID-19 mitigation strategies.

#### Are national trends and government policies more important in determining adolescent well-being at certain points in the pandemic compared to others?

A second and related question in popular discourse around COVID-19 that can be informed by the *who* and *when* results from the current study is whether national trends and government policies are more important in determining adolescent life disruption at some time points during the pandemic than other time points (Hale et al., 2021; Samji et al., 2022). We found some evidence that national trends and government COVID-19 policies may be especially important in predicting adolescent life disruption at the beginning of the COVID-19 pandemic. For instance, lower national death rates predicted *who* among adolescents experienced less COVID-19 life disruption during the first 6 months of the pandemic. Additionally, during the first 6 months of the pandemic, *when* adolescents resided in nations that had especially stringent measures of COVID-19 mitigation (compared to the rest of their pandemic response) they experienced less COVID-19 disruption. Moreover, during the first 6 months of the pandemic, *when* adolescents were more confident in their government’s response to the pandemic (compared to their own level of confidence throughout the rest of the pandemic), they experienced less COVID-19 disruption. All three of these protective effects dissipated after the first 6 months of the COVID-19 pandemic passed (i.e., after September 2020).

These results may suggest that national trends and government responses to COVID-19 were especially critical in determining adolescent well-being in the pandemic’s first 6 months. When it comes to preventing adolescent life disruption, governments may be right to heed WHO epidemiologist Dr Michael Ryan’s now-infamous wise words at the beginning of the pandemic: “You need to be coordinated. Be fast, have no regrets, you must be the first mover. If you need to be right before you move you will never win” (Sarukhan, 2020, p. 1).

#### Do adolescents become numb to the mounting deaths around them as the COVID-19 pandemic wears on?

A third question in popular discourse around COVID-19 that can be informed by *who* and *when* results from the current study is whether people, including adolescents, become numb to mounting death counts as the pandemic wears on (Yong, 2022). At a national level, the answer to this question depends on when in the pandemic it is asked. National death rates impacted adolescents more in the first or latest 6 months of the pandemic, but less so during the middle of the pandemic. Adolescents *who* resided in nations with higher death rates for the first 6 months of the pandemic experienced greater life disruption due to COVID-19, although this effect dissipated after the first 6 months. Moreover, from March 2022 to July 2022, *when* adolescents lived in nations that experienced death rates that were higher than typical, they also experienced greater COVID-19 life disruption. In other words, national trends in death rates appear to impact adolescent COVID-19 life disruption most during periods of rapid increase (i.e., COVID-19’s first few months) or decrease (i.e., COVID-19’s latest few months) in national death rates.

In contrast, adolescents never seemed more or less “numb” to the coronavirus deaths in their own personal environment. Adolescents *who* reported a death of someone close to them due to COVID-19 reported higher life disruption scores throughout the entirety of the pandemic, regardless of when the death occurred. Taken together, these findings seem to indicate that deaths of those close to adolescents due to COVID-19 disrupt adolescents’ lives during the entirety of the pandemic, whereas higher national death rates disrupt adolescents’ lives when they rapidly wax or wane.

#### How important are changes in adolescent behavior problems over the course of the COVID-19 pandemic?

A fourth question in popular discourse around COVID-19 that can be informed by *who* and *when* results from the current study is how important changes in adolescent behavior problems over the course of the COVID-19 pandemic are. The data from our study indicate that changes in adolescent behavior problems over the course of the pandemic may be more important than commonly realized. At this point, it is well established that adolescent behavior problems increased substantially during the pandemic (Angela et al., 2022; Kauhanen et al., 2022; Manchia et al., 2022; Samji et al., 2022), but results from the current study further highlight the persistent consequences of those behavioral difficulties on adolescent well-being.

Specifically, adolescents *who* experienced higher internalizing, externalizing, or smoking problems compared to their peers experienced greater life disruption across most of the pandemic. Moreover, for any given adolescent throughout the pandemic, *when* they experienced higher internalizing or smoking problems than they typically did, they also experienced higher COVID-19-related life disruption. In other words, adolescents who experienced more behavior problems had greater life disruption during the pandemic, and even after controlling for adolescents’ overall levels of behavior problems, when adolescents experienced spikes in behavior problems during the pandemic, they reported greater life disruption. Additionally, when zero-order correlations were examined, adolescent internalizing and externalizing problems emerged as two of the three risk factors that were most highly correlated with adolescent COVID-19 life disruption. Taken together, these results indicate that behavior problems experienced during the pandemic have strong and lasting associations with adolescent life disruption. Investment in interventions to improve pandemic-era levels of adolescent behavior problems are likely to pay dividends for years to come.

In summary, disaggregating *who* and *when* effects is vital for a fuller understanding of pandemic impacts. Knowing *who* among adolescents is most at-risk for pandemic-related life disruption allows policymakers and interventionists to tailor assistance to those who might need it most. Knowing *when* over the course of a pandemic adolescents experience especially high levels of life disruption allows policymakers and interventionists to know when deploying assistance would be most important. Combining these knowledge bases allows scientists to tackle important, publicly debated questions about the pandemic’s impacts with nuance and precision (Bornstein, 2021).

### Limitations and future directions

The current study has numerous strengths. As a longitudinal, cross-cultural investigation it moves beyond cross-sectional paradigms in monocultural settings to examine trajectories of COVID-19 disruption over time. Its investigation of multiple risk factors at multiple levels of ecological context moves the field beyond examining single risk factors for COVID-19 disruption to constellations of risk factors of COVID-19 disruption. Its disaggregation of *who* and *when* effects allows for the identification of *who* among adolescents experience COVID-19 life disruption and *when* throughout the pandemic such life disruption occurs.

However, the current investigation also has several limitations, and recognition of these limitations can advance future studies and analyses. First, most of the risk factors in the current study are provided by a single reporter (although national COVID-19 death rates and national COVID-19 mitigation strategies were not based on self-reports). Therefore, associations between risk factors and COVID-19 disruption may be inflated by single-reporter bias. Future investigations could include multiple reporters (i.e., parents, teachers) of both risk factors and adolescent COVID-19 life disruption, or even examine more “objective” measures of adolescent COVID-19 life disruption, such as scholastic performance on standardized tests or early career earnings. Second, most risk factors investigated in the current study are reported with a single item. Use of these single-item measures was often necessary during the worst of the COVID-19 pandemic because such assessments needed to be delivered rapidly and completed quickly to ensure adolescent functioning could be captured in real time (Skinner et al., 2021). However, it may be that these items do not completely capture the constructs that they intended to examine, and future work could more comprehensively capture such constructs with multiple items measuring each construct. Third, although the current study samples are representative of the local geographic areas from which they are drawn, they are not nationally representative. Therefore, inferences from the current study cannot be generalized to national populations. Future work that examines nationally representative samples is vital for replicating and generalizing patterns seen in the current findings. Finally, although longitudinal in nature, the current study is observational. Therefore, true causal effects between risk factors and adolescent COVID-19 life disruption cannot be inferred.

## Conclusion

Despite these limitations, the current study has much to contribute to existing literature. Study results revealed that overall rates of adolescent COVID-19 life disruption are relatively stable and somewhat high. However, within that relatively “stable, high” trajectory, slight increases in adolescent-reported life disruption across time and culture coincided with increases in COVID-19 case counts and death rates. Societal characteristics, individual adolescent experiences with COVID-19, and adolescent behavior problems each contributed to adolescent life disruption due to COVID-19. This constellation of risk factors is best understood when broken down into risk factors that predicted *who* among adolescents experienced the most COVID-19 life disruption and risk factors that predicted *when* adolescents experienced higher COVID-19 life disruption than normal.

Adolescents who, compared to their peers, lived in nations with higher national COVID-19 death rates, lived in nations with less stringent COVID-19 mitigation strategies, who had less confidence in their government’s response to COVID-19, who complied at higher rates with COVID-19 control measures, who experienced the death of someone they knew due to COVID-19, and who experienced more internalizing, externalizing, and smoking problems experienced more life disruption due to COVID-19 during part or all of the pandemic. When, compared to their typical levels of functioning or circumstances, adolescents experienced higher national death rates (from March 2022 to July 2022), experienced less stringent COVID-19 mitigation measures (from March 2020 to September 2020), experienced less confidence in government response to the COVID-19 pandemic (from March 2020 to September 2020), complied at higher rates with COVID-19 control measures, experienced more internalizing problems, or experienced greater levels of smoking (from September 2020 to March 2022) they also experienced more life disruption due to COVID-19.

These sets of findings answer important questions about whether COVID-19 mitigation strategies impacted adolescent well-being (the presence of such policies protected adolescents against life disruption, but adolescent compliance with such policies eventually led to greater life disruption as the pandemic wore on), whether government policies and national trends protected adolescent well-being (they did, especially in the first 6 months of the pandemic), whether adolescents became numb to the deaths around them due to COVID-19 (never on a personal level, perhaps in the middle of the pandemic on a national level), and the extent to which behavior problems impacted adolescent functioning during COVID-19 (very extensively, with persistent effects throughout the pandemic). Collectively, these findings might provide new insights that policymakers, interventionists, and developmental scientists can use to target and roll out interventions to promote adolescent well-being and end the high levels of life disruption adolescents in cultures around the world experienced during the COVID-19 pandemic.

## Supplementary Material

1

## Figures and Tables

**Table 1.** Descriptive statistics for demographics by cultural group

Group	*N*	Adolescent gender (% girls)	Adolescent age	Parents’ education (# of years of education completed by most educated parent)

Whole sample	1082	52%	19.98 (1.23)	14.43 (4.26)

Chongqing, China	110	53%	17.77 (0.39)	N/A

Medellín, Colombia	80	51%	19.75 (0.61)	11.55 (5.56)

Naples, Italy	82	59%	20.94 (0.36)	12.99 (4.58)

Rome, Italy	105	48%	20.74 (0.79)	14.94 (4.34)

Zarqa, Jordan	100	52%	19.11 (0.31)	14.97 (2.55)

Kisumu, Kenya	88	60%	21.04 (0.89)	13.70 (3.61)

Manila, Philippines	86	49%	19.93 (0.45)	15.10 (4.09)

Trollhättan, Sweden	88	53%	19.71 (0.61)	15.48 (2.64)

Chiang Mai, Thailand	91	53%	18.80 (0.52)	13.98 (4.22)

U.S. Black	86	51%	21.06 (0.64)	14.66 (2.52)

U.S. Latino	76	55%	20.75 (0.73)	11.97 (4.00)

U.S. White	90	42%	21.17 (0.54)	18.64 (3.36)

**Table 2.** Descriptive statistics for main study variables

Variable	*M* or *%*	SD

COVID-19 death rate per 100,000 people	36.58	34.62

Stringency of COVID-19 mitigation strategies (range: 0–100)	56.16	14.89

Confidence in government response to COVID-19 (range: 1–4)	2.58	0.98

Compliance with COVID-19 control measures (range: 1–4)	3.51	0.69

Know someone who died due to COVID-19 (% of sample who knew someone)	9.66%	29.55%

Hope of COVID-19 pandemic ending (range: 1–3)	3.22	0.80

Internalizing problems	0.00	1.00

Externalizing problems	0.00	1.00

Alcohol use	0.00	1.00

Smoking	0.00	1.00

COVID-19 related life disruption (range: 1–10)	6.26	2.47

*Note.* Internalizing Problems, Externalizing Problems, Alcohol Use, and Smoking were all standardized to ensure the mean was 0 and standard deviation was 1.

**Table 3.** Correlations among main study variables

Variable	1	2	3	4	5	6	7	8	9	10	11

1. COVID-19 death rate per 100,000 people	1.00										

2. Stringency of COVID-19 mitigation strategies	.22*	1.00									

3. Confidence in government response to COVID	−.14*	.04*	1.00								

4. Compliance with COVID-19 control measures	−.04*	.13*	.18*	1.00							

5. Know someone who died due to COVID-19	.01	−.13*	.00	−.04*	1.00						

6. Hope of COVID-19 pandemic ending	−.12*	.06*	.43*	.25*	−0.03	1.00					

7. Internalizing problems	.07*	.01	−.15*	.04*	.00	−.11*	1.00				

8. Externalizing problems	.09*	.02	−.13*	−.06*	.04*	−.15*	.54*	1.00			

9. Alcohol use	.07*	−.05*	−.01	−.06*	.01	.02	.07*	.12*	1.00		

10. Smoking	.07*	−.02	−.02	−.02	−.01	−.07*	.13*	.18*	.40*	1.00	

11. COVID-19 related life disruption	.05*	−.06*	−.21*	.05*	.07*	−.07*	.21*	.21*	.01	.09*	1.00

*Note.* This correlation table was derived before all predictors were disaggregated into between-person “who” and within-person “when” effects.

* *p* < .05.

**Table 4.** Overall trajectory of adolescent life disruption due to COVID-19 and differences by culture

Cultural group	*B*	*SE*	*p*

*Overall sample trajectory*			

Intercept	**6.09**	**0.11**	**<.01**

Linear slope	**0.38**	**0.10**	**<.01**

Quadratic slope	**−0.10**	**0.23**	**<.01**

*Culture-specific effects on intercept*			

Chongqing, China	**−2.30**	**1.00**	**.02**

Medellín, Colombia	**−1.98**	**0.63**	**<.01**

Naples, Italy	**−1.34**	**0.55**	**.01**

Rome, Italy	**−0.52**	**0.27**	**.05**

Zarqa, Jordan	0.14	0.65	.83

Kisumu, Kenya	**3.70**	**1.93**	**.05**

Manila, Philippines	0.07	0.36	.84

Trollhättan, Sweden	0.30	0.35	.39

Chiang Mai, Thailand	0.32	0.31	.31

U.S. Black	0.64	0.52	.21

U.S. Latino	**1.28**	**0.44**	**<.01**

U.S. White	**1.30**	**0.35**	**<.01**

*Culture-specific effects on linear slope*			

Chongqing, China	−1.13	1.06	.29

Medellín, Colombia	**2.13**	**0.54**	**<.01**

Naples, Italy	**1.22**	**0.49**	**.01**

Rome, Italy	−0.10	0.26	.69

Zarqa, Jordan	0.89	0.61	.15

Kisumu, Kenya	−2.41	1.35	.07

Manila, Philippines	**0.88**	**0.45**	**.05**

Trollhättan, Sweden	−0.04	0.44	.93

Chiang Mai, Thailand	0.50	0.29	.08

U.S. Black	**−1.06**	**0.47**	**.02**

U.S. Latino	**−1.07**	**0.41**	**<.01**

U.S. White	**−0.75**	**0.35**	**.03**

*Culture-specific effects on quadratic slope*			

Chongqing, China	0.38	0.25	.13

**Medellín, Colombia**	**−0.57**	**0.10**	**<.01**

Naples, Italy	−0.16	0.10	.09

Rome, Italy	0.02	0.06	.72

Zarqa, Jordan	−0.22	0.12	.06

Kisumu, Kenya	0.42	0.22	.06

Manila, Philippines	−0.17	0.14	.21

Trollhättan, Sweden	0.06	0.11	.57

Chiang Mai, Thailand	−0.04	0.07	.52

**U.S. Black**	**0.26**	**0.10**	**<.01**

U.S. Latino	0.17	.09	.07

U.S. White	0.10	.08	.19

*Note.* Each culture-specific effect estimate can be interpreted as the difference in the culture’s intercept/linear slope/quadratic slope from the overall sample trajectories’ intercept/linear slope/quadratic slope. Bolded values are significant at *p* < .05.

**Table 5.** Primary model predicting adolescent life disruption due to COVID-19 from risk factors

	B	SE	p

*Baseline model*			

Intercept	**6.09**	**0.11**	**<.01**

Linear slope	**0.38**	**0.10**	**<.01**

Quadratic slope	**−0.10**	**0.23**	**<.01**

*“Who” between-person effects on adolescent life disruption due to COVID-19 intercept*			

COVID-19 death rate per 100,000 people	**0.02**	**0.01**	**<.01**

Stringency of COVID-19 mitigation strategies	**−0.04**	**0.01**	**<.01**

Confidence in government response to COVID-19	**−0.37**	**0.10**	**<.01**

Compliance with COVID-19 control measures	0.13	0.18	0.47

Know someone who died due to COVID-19	**0.44**	**0.17**	**<.01**

Hope of COVID-19 pandemic ending	0.21	0.12	0.09

Internalizing problems	**0.77**	**0.11**	**<.01**

Externalizing problems	**0.27**	**0.11**	**.02**

Alcohol use	−0.04	0.09	.67

Smoking	−0.07	0.16	.67

Cultural group	**0.30**	**0.06**	**<.01**

Adolescent gender	−0.15	.13	.22

Adolescent age	0.07	0.08	.35

Parents’ education	**−0.07**	**0.03**	**<.01**

*“Who” between-person effects on adolescent life disruption due to COVID-19 linear slope*			

COVID-19 death rate per 100,000 people	**−0.03**	**0.01**	**<.01**

Compliance with COVID-19 control measures	**0.12**	**0.06**	**.05**

Smoking	**0.34**	**0.15**	**.02**

Cultural group	**−0.34**	**0.05**	**<.01**

Parents’ education	**0.06**	**0.02**	**.02**

*“Who” between-person effects on adolescent life disruption due to COVID-19 quadratic slope*			

COVID-19 death rate per 100,000 people	**0.01**	**0.00**	**<.01**

Smoking	**−0.08**	**.03**	**.01**

Cultural group	**0.07**	**0.01**	**<.01**

Parents’ education	**−0.01**	**0.01**	**<.01**

*“When” within-person effects on adolescent life disruption due to COVID-19*			

COVID-19 death rate per 100,000 people	0.01	0.02	.36

COVID-19 death rate per 100,000 people * time	**−0.03**	**0.01**	**.05**

COVID-19 death rate per 100,000 people * time^2^	**0.01**	**0.00**	**<.01**

Stringency of COVID-19 mitigation strategies	**−0.13**	**0.04**	**<.01**

Stringency of COVID-19 mitigation strategies * time	**0.14**	**0.04**	**<.01**

Stringency of COVID-19 mitigation strategies * time^2^	**−0.03**	**0.01**	**<.01**

Confidence in government response to COVID-19	0.27	0.19	.15

Confidence in government response to COVID-19 * time	**−0.55**	**0.20**	**<.01**

Confidence in government response to COVID-19 * time^2^	**0.11**	**0.05**	**.02**

Compliance with COVID-19 control measures	**0.18**	**0.08**	**.03**

Hope of COVID-19 pandemic ending	0.12	0.08	.11

Internalizing problems	**0.23**	**0.07**	**<.01**

Externalizing problems	0.00	0.06	0.95

Alcohol use	−0.07	0.06	.21

Smoking	**0.16**	**0.06**	**<.01**

*Note.* “Baseline Model” values are intercept, slope, and quadratic slope scores for the average adolescent in the data set before accounting for any “who” or “when” effects or covariates. Bolded values are significant at *p* < .05.

## Footnotes

1 We define the individuals in our sample as “adolescents” instead of “young adults” to align with contemporary developmental science’s definition of adolescence, which is now thought to extend from age 10 to 24 (Sawyer et al., 2018).
