# Supplementary material for: How adolescents’ lives were disrupted over the course of the COVID-19 pandemic: A longitudinal investigation in 12 cultural groups in 9 nations from March 2020 to July 2022
Source: Dev Psychopathol. Author manuscript; Available in PMC 2025 Aug 1. (PMC11272905; doi:10.1017/S0954579423001621)
Supplement: 1 [file NIHMS1952450-supplement-1.pdf]

# EXPERIENCES RELATED TO COVID-19 (EC19-R) (Father/Mother/Youth)

Thinking about your thoughts, feelings, and behaviors around the COVID-19 (coronavirus) illness, please answer the following questions, focusing on your thoughts and behaviors in the last three months.

Scale:
1 = Strongly disagree
2 = Somewhat disagree
3 = Somewhat agree
4 = Strongly agree

1. We recognize that many local, state, and federal government agencies are involved in the response to COVID-19. Balancing your perspective on all of these agencies…I am confident the government is handling the COVID-19 response in the best possible manner.... **EC19R_1_F/EC19R_1_M/EC19R_1_Y**
2. I am hopeful that the COVID-19 virus will resolve over time and I have a good outlook toward the future. ........................................................................................... **EC19R_2_F/EC19R_2_M/EC19R_2_Y**
3. I complied with the rules and suggestions of the government and health care system to try to contain the virus. ............................................................................... **EC19R_3_F/EC19R_3_M/EC19R_3_Y**

COVID-19 may have many impacts on you and your family life. Thinking about your life right now and in the last 3 months, how has the COVID-19 pandemic affected each of the following?
Scale:
1 = Made it a lot better
2 = Made it a little better
3 = No change
4 = Made it a little worse
5 = Made it a lot worse

4. Parenting (if you have a child) ............................................................ **EC19R_4_F/EC19R_4_M/EC19R_4_Y**
5. How family members get along with each other.................................. **EC19R_5_F/EC19R_5_M/EC19R_5_Y**
6. Your exercise......................................................................................... **EC19R_6_F/EC19R_6_M/EC19R_6_Y**
7. Your eating ........................................................................................... **EC19R_7_F/EC19R_7_M/EC19R_7_Y**
8. Your sleep.............................................................................................. **EC19R_8_F/EC19R_8_M/EC19R_8_Y**
9. Your feelings of anxiety ........................................................................ **EC19R_9_F/EC19R_9_M/EC19R_9_Y**
10. Your feelings of depression/sadness............................................. **EC19R_10_F/EC19R_10_M/EC19R_10_Y**
11. Your anger ...................................................................................... **EC19R_11_F/EC19R_11_M/EC19R_11_Y**

12. Your getting into arguments........................................................ **EC19R_12_F/EC19R_12_M/EC19R_12_Y**
13. Spending time doing fun things with family ................................ **EC19R_13_F/EC19R_13_M/EC19R_13_Y**
14. Spending time doing fun things with friends................................ **EC19R_14_F/EC19R_14_M/EC19R_14_Y**
15. Your social support network ....................................................... **EC19R_15_F/EC19R_15_M/EC19R_15_Y**
16. Your smoking.................................................................................. **EC19R_16_F/EC19R_16_M/EC19R_16_Y**
17. Your alcohol use............................................................................. **EC19R_17_F/EC19R_17_M/EC19R_17_Y**
18. Your illicit drug use........................................................................ **EC19R_18_F/EC19R_18_M/EC19R_18_Y**
19. Your working conditions................................................................. **EC19R_19_F/EC19R_19_M/EC19R_19_Y**
20. Your income.................................................................................... **EC19R_20_F/EC19R_20_M/EC19R_20_Y**

Scale:
0 = No
1 = Yes

21. Have you been tested for the COVID-19 virus?............................ **EC19R_21_F/EC19R_21_M/EC19R_21_Y**
22. Have you tested positive for the COVID-19 virus? ...................... **EC19R_22_F/EC19R_22_M/EC19R_22_Y**
23. Do you have a close friend or loved one who has tested positive for the COVID-19 virus? ........................................................................................................ **EC19R_23_F/EC19R_23_M/EC19R_23_Y**
24. This may be a difficult question, but has someone close to you lost their life due to the COVID-19 ........................................................................................................ **EC19R_24_F/EC19R_24_M/EC19R_24_Y**
25. Please rate how much the COVID-19 outbreak has been disruptive to you personally. Think about your daily routines, work, and family life......................................................... **EC19R_25_F/ EC19R_25_M/ EC19R_25_Y**

| 1 | 2 | 3 | 4 | 5 | 6 | 7 | 8 | 9 | 10 |
|---|---|---|---|---|---|---|---|---|---|
| Not at all disruptive | | | | somewhat disruptive | | | | extremely disruptive | |
